# Position of human blood group O(H) and phenotype‐determining enzymes in growth and infectious disease

**DOI:** 10.1111/nyas.13694

**Published:** 2018-05-12

**Authors:** Peter Arend

**Affiliations:** ^1^ Department of Medicine Philipps University Marburg, Marburg/Lahn, Germany. Gastroenterology Research Laboratory, College of Medicine, University of Iowa, Iowa City, Iowa. Research Laboratories, Chemie Grünenthal GmbH Aachen Germany

**Keywords:** blood group O position, complementary pathogen, phenotypic glycosidic accommodation, hybrid Tn antigen, nonimmune immunoglobulin M (IgM)

## Abstract

The human ABO(H) blood group phenotypes arise from the evolutionarily oldest genetic system found in primate populations. While the blood group antigen A is considered the ancestral primordial structure, under the selective pressure of life‐threatening diseases blood group O(H) came to dominate as the most frequently occurring blood group worldwide. Non‐O(H) phenotypes demonstrate impaired formation of adaptive and innate immunoglobulin specificities due to clonal selection and phenotype formation in plasma proteins. Compared with individuals with blood group O(H), blood group A individuals not only have a significantly higher risk of developing certain types of cancer but also exhibit high susceptibility to malaria tropica or infection by *Plasmodium falciparum*. The phenotype‐determining blood group A glycotransferase(s), which affect the levels of anti‐A/Tn cross‐reactive immunoglobulins in phenotypic glycosidic accommodation, might also mediate adhesion and entry of the parasite to host cells via trans‐species *O*‐GalNAc glycosylation of abundantly expressed serine residues that arise throughout the parasite's life cycle, while excluding the possibility of antibody formation against the resulting hybrid Tn antigen. In contrast, human blood group O(H), lacking this enzyme, is indicated to confer a survival advantage regarding the overall risk of developing cancer, and individuals with this blood group rarely develop life‐threatening infections involving evolutionarily selective malaria strains.

## Graphical abstract

The article substantiates the position of the human blood group O during the evolution of primates by its central function in defense against both life‐threatening infections by an evolutionarily selective eukaryotic pathogen and the early stages of cancer development, and discusses the key role played by phenotype‐determining enzymes.

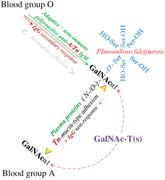

## Introduction

**Figure 1:**
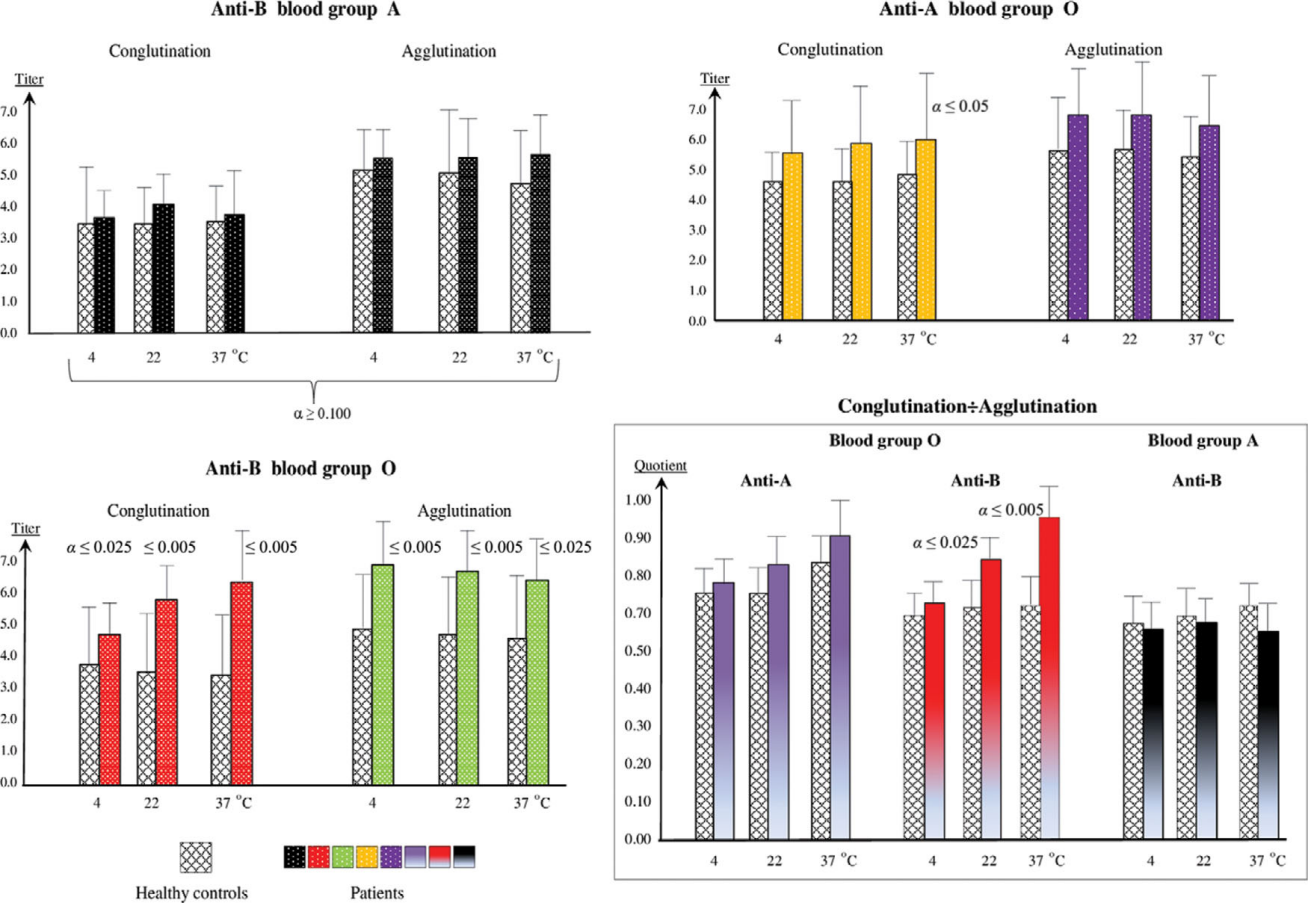
Selective immune response of blood group O individuals to increased enteral absorption: spontaneous variations in the production of anti‐A/B isoagglutinins in 21 individuals suffering from ulcerative colitis (blood group A = 11, blood group O = 10), and in 42 normal persons (blood group A = 22, blood group O = 20). The degree of immunization and dominance of the 7S (IgG) conglutinating and 19S (IgM) agglutinating immunoglobulins were determined by measuring conglutinin and agglutinin titers and their quotients in triple serum samples at three different temperatures under double‐blind conditions. The number of the respective dilution stage was used for the evaluation; this number corresponds to the negative logarithm of base 2 of the dilution. By means of the nonparametric Wilcoxon signed‐rank test, the anti‐B‐reactive IgG and IgM levels in blood group A plasma remained within normal range, whereas the blood group O patients exhibited the statistically significant stimulation of an IgG conglutinating and IgM agglutinating anti‐B response as well as an asymmetrically cross‐reactive, less pronounced anti‐A specific IgG response, and the IgG/IgM quotients indicate the predominance of the IgG class. The figure is reconstructed from the data published in Arend and Fehlhaber.^6^

The human gut microbiome has been a subject of comprehensive research for decades, and numerous reviews have discussed various aspects of host–microbe interactions in the context of health and disease. In terms of host genetics and immunity,^1^, ^2^, ^3^ naturally occurring immunoglobulins arise from various sources via different molecular pathways, and the ABO blood group is among the genetically determined host factors that modulate the composition of the human intestinal microbiota.^4^ Specifically, the microbiota of blood group B individuals differs from those of individuals with non‐B antigen phenotypes, for example, showing higher diversity in the *Eubacterium rectale* and *Clostridium leptum* groups. However, when the adaptive production of anti‐blood group B‐reactive immunoglobulins, which occurs in White Leghorn chickens fed a diet containing *Escherichia coli* O86:B7 lipopolysaccharide,^5^ was demonstrated for the first time to occur spontaneously in humans,^6^ this form of isoagglutinin production was documented exclusively for the histo (blood) group O(H). Bacterial endotoxins nonspecifically stimulate the formation of all immunoglobulins, but prokaryotic blood group A/B‐like antigenic structures appear to induce cross‐reactive anti‐A/B immunoglobulin G^7^, ^8^, ^9^ that arise in neither blood group A nor B individuals but occur predominantly or exclusively in blood group O(H) due to clonal selection. Consequently, using the nonparametric Wilcoxon signed‐rank test in patients suffering from ulcerative colitis, which causes increased enteral absorption, the minimally and likely nonspecifically elevated levels of anti‐B‐reactive 7S (IgG) and 19S (IgM) in blood group A plasma were found to remain within the normal range; additionally, a statistically significant increase in anti‐B‐reactive IgG and IgM immunoglobulins involving less pronounced, asymmetrically cross‐reactive anti‐A‐specific IgG was detected exclusively in blood group O(H) plasma, and the IgG/IgM quotients showed predominance of the IgG class (Fig. 1). These early, limited observations are consistent with later investigations performed with current experimental tools. For example, Stussi *et al*.^10^ detected anti‐A/B cross‐reactive IgG in 89% of blood group O(H) sera and anti‐B‐reactive IgG in 4% of blood group A sera, and vaccination with pneumococcal polysaccharides exclusively elicited anti‐A/B cross‐reactive IgG but did not affect preexisting anti‐A/B‐reactive IgM levels.^11^ While the danger theory^12^ suggests that there is no adaptive immunity without innate immunity, the bulk of human immunity is not acquired during a single human lifetime but is considered to arise predominantly from evolutionary memory and survival mechanisms. Thus, an inborn origin has been postulated to explain isoagglutinin production in non‐O blood groups.^13^, ^14^ In addition to adaptive, cross‐reactive anti‐A/B production, which is mainly restricted to blood group O(H) individuals, the majority of anti‐A/B immunoglobulins, especially the classic complement‐binding anti‐A‐ and anti‐B‐reactive isoagglutinins, measured between 22 and 24 °C, are not controlled by clonal selection and primarily arise independent of any blood group. These immunoglobulins result from a polyreactive, nonimmune, germline‐encoded IgM molecule that is released after germ cell maturation and cell renewal, and undergoes phenotype‐specific glycosidic accommodation of plasma proteins in the non‐O blood groups.^13^, ^15^ It is proposed that depending on the quality of glycosylation, the degree of glycosylation of an immunoglobulin is inversely proportional to its reactivity. While any phenotype‐directed autoreactivity, primarily exerted by germline‐encoded antibodies, may automatically be neutralized during the course of phenotype formation in normal condition, the dynamic glycosylation of immunoglobulins presumably plays a key role in physiology and mainly occurs via *N*‐linkages. Nevertheless, a proteomics analysis of *O*‐GalNAc glycosylation in human serum identified 407 intact *O*‐GalNAc glycopeptides from 93 glycoproteins.^16^ Thus, in blood group A and B individuals, phenotype‐determining GalNAc‐ or d‐Gal glycosylations of plasma proteins are, aside from prevailing *N*‐glycosylations, hypothetically associated with mucin‐type formation, utilizing *O*‐linkages to functional serine/threonine or tyrosine residues from the Fc (see Ref. 130) and/or V (see Ref. 17) region of the ancestral IgM molecule. The terminal serine appears to be the crucial structure,^18^ and any reduction or exclusion of IgM anti‐self‐reactivity thereby achieved necessarily impairs adaptive and innate defense activities.^15^ The key molecule in this phenotypic accommodation might be α2‐macroglobulin, which is considered to be an evolutionarily conserved arm of the innate immune system.^19^ α2‐Macroglobulin is functionally strongly connected to the structurally related IgM molecule, as becomes evident from adhesion of the parasite *Plasmodium falciparum* to the host cell surface in severe malaria disease,^20^ and exhibits ABO(H) blood group reactivity in strict correlation with the cell surface.^21^, ^22^ Consequently, in blood group O(H) individuals, the nonimmune and adaptive anti‐A (always with Tn cross‐reactive) IgM/IgG^15^ levels remain unaffected and are still involved in internal and external immune defense. Moreover, the binding of nonimmune IgM to the trans‐species, blood group A‐like or Tn (T‐nouvelle) antigen (GalNAc‐1‐*O*‐Ser/Thr)^23^ and to the B‐like or T (Thomsen‐Friedenreich) antigen (Gal‐1‐3GalNAc‐1‐*O*‐Ser/Thr),^24^ might initiate a secondary response involving the formation of anti‐A/Tn and B/T IgG activities, with T cell and natural killer cell activation,^25^, ^26^ which in the non‐O blood groups A, B, and AB is affected by glycosidic, phenotypic accommodation. This appears to be a general principle that not only may contribute to the increased susceptibility of non‐O blood groups to cancer development but even explains the varying susceptibilities of the different ABO(H) phenotypes to special infectious diseases via trans‐species molecular complementarity.

## Molecular relationship of human ABO(H) blood group phenotype evolution and development to growth and malaria infections

Clonal selection and the principle of glycosidic, phenotypic accommodation explained above have been postulated to contribute to the increased risk of human blood group A individuals developing different types of cancer,^15^ and the naturally occurring anti‐A‐reactive isoagglutinins and anti‐Tn‐cross‐reactive antibodies clearly interact with tumor‐associated *O*‐glycan structures produced in pancreatic cancer.^27^ In a recent cancer risk cohort study, Sun *et al*.^28^ described a statistically significant risk of stomach cancer and pancreatic cancer in blood group A individuals compared with blood group O(H) individuals, while kidney cancer risk was inversely associated with blood type AB. A comprehensive study by Vasan *et al*.^29^ showed positive associations in blood group A individuals with cancer of the pancreas, breast, salivary glands, mouth, stomach, and chronic lymphatic leukemia and in blood group B individuals with cancer of the corpus uteri and the bladder; however, inverse associations were observed in blood group A individuals for pharyngeal cancer, esophageal adenocarcinoma, and small intestinal cancer and in blood group B individuals for pleural mesothelioma and myelomas. In two smaller studies, regarding the O/O and A/A genotypes, the above positive associations became even clearer in both cancer of the stomach^30^ and that of the pancreas^31^ and were finally considered to be established. Thus, blood group A individuals might be burdened with an overall increased risk of developing cancer compared with individuals exhibiting blood group O(H), which is currently thought to confer a survival advantage compared with non‐O blood groups.^32^, ^33^

Considering that a species barrier may be overcome by interspecies glycosylations^34^ or pathogen mimicry, and ABO(H) glycotransferases accomplish the cross‐species transmission of *O*‐glycans in infectious diseases,^35^ the proposed principle of glycosidic, phenotypic accommodation may explain the pronounced susceptibility of non‐O blood group individuals to the life‐threatening infections characteristic of classic malaria tropica, which is caused by *P. falciparum*.^36^ While malaria tertiana or infection by *Plasmodium vivax* is the best‐documented type of malaria,^37^ *P. falciparum* causes the most severe and frequently encountered type of the disease.

## The susceptibility of non‐O blood group individuals to the infection by *P. falciparum* resides in molecular complementarity between the host and parasite

**Figure 2:**
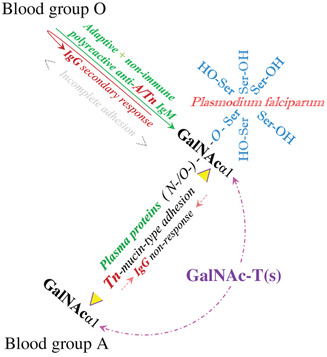
Complementarity and adhesion of mammalian structures to a pathogen exclude the formation of adaptive and innate antibody reactivity against the resulting hybrid antigen. The parasite *Plasmodium falciparum* exhibits molecular complementarity to blood A synthesis by serine repeat antigen (SERA), exhibiting a surplus of serine residues, which may function as acceptor sites on the surface of inoculated sporozoites for the soluble blood group A‐determining plasma transferase α1‐3‐*N*‐GalNAc‐T, hypothetically synthesizing a trans‐species, hybrid A‐like, Tn‐mucin‐type GalNAc‐1‐*O*‐Ser/Thr‐R structure. Blood group A cannot respond by immunoglobulin G (IgG) antibodies to A or A‐like cross‐reactive Tn structures due to clonal selection, while the nonimmune IgM undergoes phenotypic accommodation and, moreover, becomes involved in the adhesion mechanism by its Fc‐region^66^ via *O*‐GalNAc glycosylation of germ‐line serine residues.^18^ Individuals with blood group O(H), who lack this enzyme, may not develop this hybrid antigen and maintain the antibodies against it, while the parasite does not completely become attached to the cell membranes of the host.

**Figure 3:**
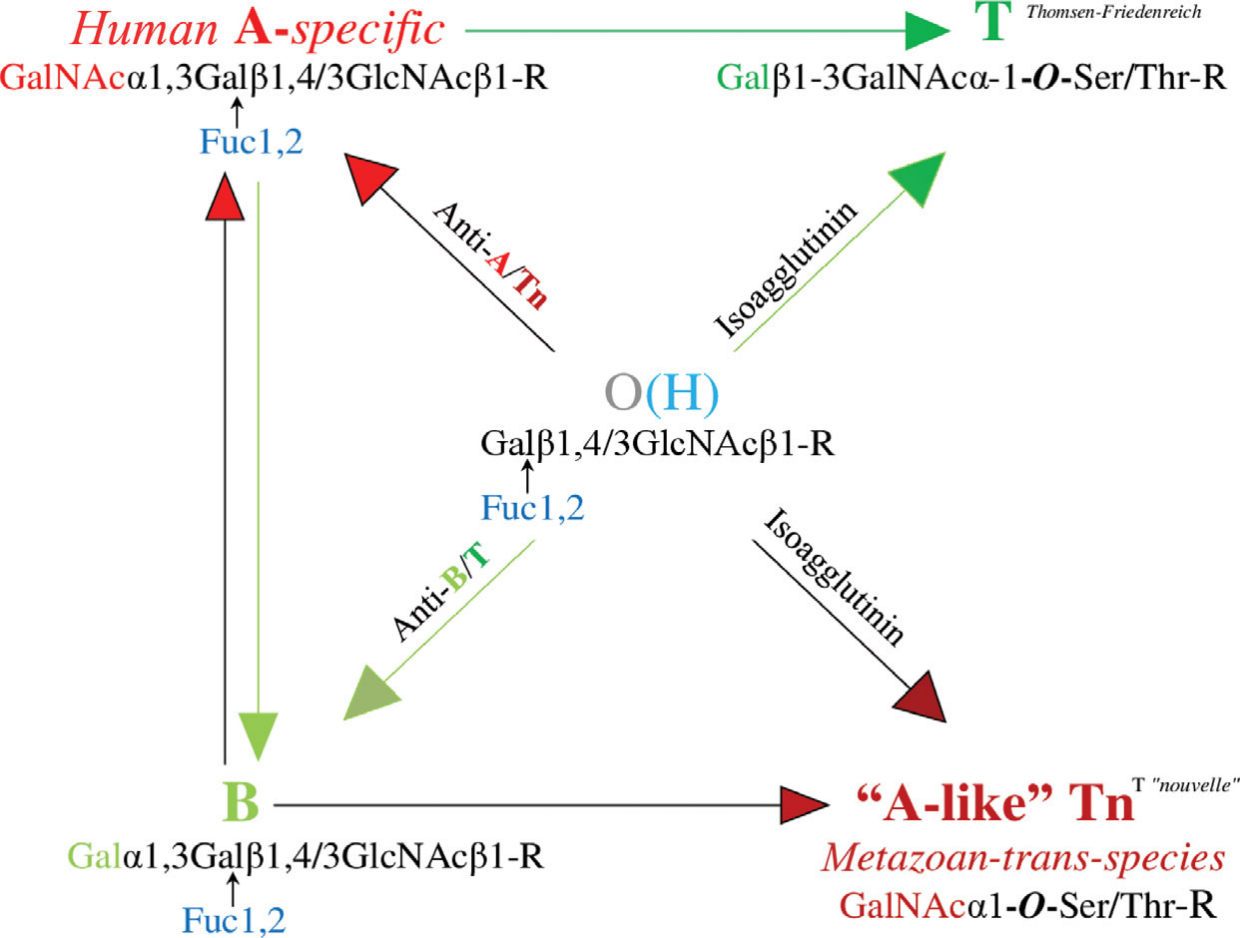
The central immunological position of blood group O(H) is evident based on its comprehensive presentation of both adaptive and germline‐encoded natural antibodies against all mature A and B glycans and their cross‐reactive developmental structures Tn and T. Human A‐specific (A‐allelic) glycosylation and trans‐species A‐like Tn formation are developmentally connected via the formation of cross‐reactive anti‐A/Tn isoagglutinin. According to Hofmann *et al*.,^27^ blood group O(H) sera bind to both Tn and T antigens, and the anti‐A isoagglutinin levels in blood group O(H) and blood group B sera are associated with an anti‐Tn antibody that does not react with blood group B red cells or T glycoconjugates. By contrast, the anti‐B antibodies of blood group A sera and blood group O(H) sera bind to B and T glycoconjugates but not to A or Tn glycoconjugates. This figure was constructed according to Arend.^15^

According to a new coalescence analysis performed by Yamamoto *et al*.,^38^ blood group antigen A was again confirmed as the primordial blood group structure and came first in the evolution. After millions of years, this group mutated in B, while a human‐specific diagnostic deletion for blood group O (O01) haplotype) was found in Neandertal individuals,^39^ and finally blood group O(H) became the most common blood group worldwide. There are many possible explanations for this phenomenon. Over millions of years, an internal selection process might occur via a currently discussed survival advantage of the overall risk of developing cancer and silent ABO(H) incompatible pregnancies, almost restricted to the blood group O(H), in which maternal IgG antibodies pass through the placenta to the fetal circulation, and cause more or less silent abortions. However, in view of the global distribution of the ABO(H) blood groups, an evolutionarily selective infectious disease like malaria appears to be the major reason. While the life of malaria species depends exclusively on the biological altruism of higher eukaryotic species, in the case of malaria tropica this trans‐species altruism is predominantly present in the human non‐O blood groups with self‐destructive behavior, and especially the blood group A phenotype is highly burdened by life‐threatening infection. The evolution of humankind most likely occurred in Africa,^40^ where infections by *P. falciparum* or malaria tropica are endemic, while the non‐O blood groups A, B, and AB are highly susceptible to these infections, with the blood group A appearing to be more affected by the life‐threatening form of this disease than blood group B but due to the available numbers is also best studied and compared with blood group O(H).^41^ Consequently, blood group O(H) came to dominate, and its current global distribution is consistent with a selection pressure by *P. falciparum* in favor of group O(H) individuals, who have a survival advantage in malaria‐endemic regions.^42^, ^43^ Thus, malaria tropica is currently discussed as contributing to the varying global distribution of ABO(H) blood groups in the human population.^44^ Infection by this parasite occurs mainly in tropical and subtropical areas of South America, Africa, and south‐east Asia,^45^ although episodes of global distribution, involving life‐threatening diseases, are a widely discussed problem.^46^ Statistically significant life‐threatening infections are diagnosed in blood group A,^47^ B, and AB^48^ individuals compared with those with blood group O(H), although in areas where this type of malaria is endemic, blood group O(H) individuals may represent the largest group of gametocyte carriers, who sometimes even suffer from mild disease.^43^ *P. falciparum* differs from the other human malaria species in that infected red blood cells (RBCs) do not remain in the circulation for the entire life cycle; instead, when young parasites mature to the trophozoite stage, infected RBCs adhere to endothelial cells in the microcirculation.^49^ This phenomenon, termed “sequestration,” appears to be another characteristic of life‐threatening disease resulting from *P. falciparum* infection, in addition to RBC rosette formation. The parasite binds by the PfEMP1 protein (*P. falciparum* erythrocyte membrane protein 1)^50^ to RBC surfaces via the common *Duffy* binding‐*like* domain, and its binding to the RBCs of non‐O blood groups^41^ is statistically significantly increased when compared with those of group O(H). This binding even correlates with variants of ABO glycosyltransferase genes^131^ and suggests that the soluble form of these enzymes themselves are engaged in the binding process. In fact, because the RBC membrane lacks detectable expression of the blood group A‐ and B‐determining transferases, it is assumed that the binding between host and pathogen is basically accomplished by plasma proteins and soluble transferring enzymes. Indeed, soluble transferases, which likely catalyze the formation of soluble blood group A/B structures and phenotype formation of plasma proteins^15^ (and occurring independently of the secretor status^51^, ^52^) may also perform the attachment of the inoculated plasmodium sporozoites to the cell membranes of the host. A functional soluble blood group A‐transferring serum transferase has been described by Nagai *et al*.,^53^ who transferred UDP‐GalNAc to blood group O RBCs *in vitro*. Although a transfer of this molecule to a pathogen has never been reported before, the coincidence of a statistically significant occurrence of life‐threatening infections by *P. falciparum* with the synthesis of blood group A‐ and B‐determining transferases in nonblood group O populations strongly suggests that trans‐species glycosylation of blood group A and B glycans plays a key role in this disease. Breaking the species barrier, transglycosylation and transpeptidation may be fundamental in the development of infectious diseases. According to Varki and Lowe,^34^ viruses perform the glycosylations of their proteins by utilizing the host cell machinery, and pathogens invading multicellular animals may decorate themselves with structures that appear to be identical to those found on their host cell surfaces. Kreisman *et al*.^54^ argued that a pathogen seeking to bind to cell membranes of the host may first encounter the specific glycans attached to soluble mucinous structures. In fact, while mucin‐type *O*‐GalNAc glycosylation likely plays a major role in cell adhesions,^55^ soluble blood group A‐ and B‐determining enzymes hypothetically initiate the synthesis of adhesion molecules via heterologous mucin‐type *O*‐glycosylation of the parasite's serine repeat antigen (SERA); that is, a serine‐rich protein providing numerous *N*‐ and *O*‐glycosidic sites. This protein primarily appears during the intraerythrocytic stages of the parasite, while serine residues are expressed at all stages of the parasite's life cycle, and even occur on the surface of inoculated sporozoites, which are proteolytically expressed by a serine protease.^56^ The SERAs are encoded from a multigene protease family, common to all *Plasmodium* species^57^, ^58^ but occurring in different types, from which the serine‐type SERA SERA5 and cysteine‐type SERA SERA6 show the highest levels in *P. falciparum*.^59^, ^60^ According to a recent study, SERA5 has been discussed to have no enzymatic role^61^ during the blood‐stage growth of *P. falciparum*, and although obviously regulating the kinetics and efficiency of malaria parasite invasion and egress from host erythrocytes, neither SERA5 function nor the role of its processing appear to be completely understood.^62^ Nevertheless, in view of the strong susceptibility of the humans with blood group A to infection by *P. falciparum*, SERA5 is strongly suggested to function as an acceptor in enzyme‐substrate competition between host and parasite in mucin‐type *O*‐GalNAc glycosylations. Again, as viruses are performing the glycosylations of their proteins by utilizing the host cell machinery,^34^ plasmodium species may utilize the same method (Fig. 2). Without questioning the established role of RIFINs,^44^, ^63^, ^64^, ^65^ which are discussed to be provided by the pathogen alone, a functional blood group A‐determining plasma transferase transferring the blood group A glycan may indicate an additional and/or more complex pathogenic mechanism, involving the trans‐species A‐like, Tn‐mucin‐type GalNAc‐1‐*O*‐Ser/Thr‐R structure,^23^ representing a classical adhesion molecule that promotes glycan elongations and the binding between parasite and host. Finally, the human blood group A–specific α1‐3‐*N*‐acetylgalactosaminyltransferase and blood group B‐specific α1‐3‐galactosyltransferase, expressed by plasma proteins might, together with serine/threonine kinases produced by *P. falciparum*, provide the essential metabolic condition for trans‐species synthesis of mucin‐type adhesion proteins and GalNAc‐1‐*O*‐Ser/Thr or Tn^23^ glycosylation in blood group A, and Gal‐1‐3GalNAc‐1‐*O*‐Ser/Thr or T^24^ glycosylation in the blood group B. This proposed glycosidic accommodation of plasma proteins^14^, ^15^ is not a lock‐and‐key event but a dynamic process, dominated by continuous synthesis of α2‐macroglobulin and its functional synergism with the structurally related nonimmune IgM; with this molecule being engaged in the adhesion process via its Fc region^17^, ^66^ and *O*‐glycosylation^14^, ^15^ of germ‐line serine residues,^18^ it obviously cannot induce rosetting on its own,^20^ whereas α2‐macroglobulin crosslinks multiple PfEMP1 molecules and, thus, is an integral component of rosette formation. The differences in the life cycle of the parasite during uncomplicated malaria in blood group O(H) individuals and life‐threatening disease in blood group A and B individuals remain unknown. Again, the complex infection process is presumably initiated by heterologous glycosylation, which has long been considered the major carbohydrate modification in the intraerythrocytic stage of *P. falciparum*,^67^ and *O*‐glycosylation appears to be the major form,^68^ whereas the role of the ABO(H) phenotypes has largely been ignored. The metazoan/eukaryotic lineage, which comprises the evolutionarily first protein glycosylation by GalNAc via *O*‐linkages on peptides displaying serine/threonine motifs, is characterized by mammalian embryonic stem cell fidelity in order to initiate and complete germ cell maturation. When breaking the species barrier, this glycosylation may promote the asexual reproduction of *Plasmodium* merozoites, which involves immature gamete formation, and may even explain the attachment of infected to uninfected RBCs. Indeed, rosette formation occurs predominantly with human blood group A RBCs,^41^, ^47^ while incomplete formation in blood group O(H) RBCs, as molecularly explained by Moll *et al*.,^69^ may be associated with weak disease. Serine/threonine and tyrosine kinases are exported by *P. falciparum*,^70^, ^71^ and the infected RBC membrane exhibits serine transferase activity, representing a potential antimalaria drug target during the asexual intraerythrocytic stage.^71^, ^72^ Thus, SERA, which was detected decades ago,^73^ was accepted as a putative antigen precursor, while heterologous *O*‐GalNAc glycosylation, which most likely utilizes trans‐species compatible serine positions on the surface of plasmodium sporozoites, might explain the preferred binding of special *Plasmodium* strains. In conclusion, the pronounced susceptibility of blood group A individuals to severe malaria^36^ suggests the formation of a hybrid Tn‐reactive structure, providing another molecular definition of a potential immunological (therapeutic) target, while blood group O(H) individuals, who are unable to complete this self‐destructive, likely transient, hybrid connection further maintain both natural nonimmune anti‐A/Tn IgM and IgG cross‐reactive antibodies against this deleterious connection (Figs. 2 and 3).^15^ In blood group B, the adhesion of plasmodia to RBCs presumably occurs via Gal‐1‐3GalNAc‐1‐*O*‐Ser/Thr or T^24^ glycosylation, which additionally acts also in blood group AB. In view of this concept, the weak or incomplete adhesion of the parasite on the blood group O(H) cell surfaces could raise speculation on *O*‐fucosylations, carried out by soluble ɑ1,2‐l‐fucosyltransferases on the parasite's peptide serine residues. However, incomplete adhesions may also arise via nonspecific, blood group A‐ and  B‐independent glycans, while analyses of AO exons and introns in individuals with an A‐to‐weak B phenotype have revealed a novel O1v‐A2 hybrid allele leading to missense mutations in A‐transferases and/or transferees.^74^ Since using the blood type molecular testing and genotyping in transfusion medicine, the number of weak ABO(H) alleles is still growing.^75^ Unusual O alleles, including O2, at the ABO(H) locus may be implicated in unexpected blood group phenotypes,^74^ whereas the O(H) phenotype is no longer considered a genetic entity.^76^, ^77^ It would be interesting to study the binding of *P. falciparum* to weak A alleles that appear to serologists as group O but produce irregular anti‐A_1_, whose reactivity with Tn‐structures is unknown and also arises in some individuals with blood group A_2_,^78^ which most likely results from the lack of GalNAc transferase A_1_ and exclusive encoding of a specific GalNAc transferase A_2_. This human‐intrinsic enzyme is genetically distinct from A_1_ transferase.^79^, ^80^, ^81^ Furthermore, in view of the more recent experiments by Blixt *et al*.,^82^ the chemical simplicity of the Tn antigen does not necessarily stand for a simple antigenicity, which changes with various peptide backbones. Finally, blood‐type molecular and genotyping studies on malaria patients with weak A alleles in connection with nonimmune, natural anti‐A/Tn levels potentially provide even more insight into the actual trend of ABO(H) blood group evolution.

## Induction of infectious diseases occurs via molecular complementarity between host and pathogen

The process of *P. falciparum* merozoite invasion, most likely involving SERA5, shows an intriguing connection to early metazoan growth processes, which appear to be dominated by trans‐species *O*‐GalNAc1‐*O*‐Ser/Thr‐R or Tn glycosylation.^83^, ^84^ Under normal conditions, this glycosylation is followed by rapid deglycosylation and may further be followed by the specific glycosidic construction of the A, B, and AB phenotypes in humans^15^ after zygote formation, with the exception of blood group O(H). These phenotypes arise from the evolutionarily oldest genetic system found in primate populations^85^ but always occur in critical complementarity with the metabolism and recognition tools of distinct eukaryotic and prokaryotic pathogens. The species‐independent ABO gene polymorphism has been extensively analyzed in primates,^80^, ^86^ but an understanding of the ramifications of this polymorphism has been elusive.^87^ While the pronounced susceptibility of blood group A individuals to the infection by the eukaryote *P. falciparum* appears to be established and is explainable molecularly through different models, reports describing the susceptibility of different ABO(H) blood groups to prokaryotic, viral and bacterial infections are more controversial.^88^, ^89^, ^90^ Although the phenomenon of pathogen mimicry is known for decades, there are few examples by which the complementarity between the phenotype‐determining enzymes of the host and the acceptor of the pathogen becomes molecularly evident. Focusing on the blood group O and its discussed susceptibility to infection by *Helicobacter pylori* and special strains of *Vibrio cholerae* reveals that the genetic, metabolic, and immunological relationships between host and pathogen are much more complex than those between the non‐O‐blood groups and the eukaryotic parasite *P. falciparum* or the malaria tropica disease. Whereas intestinal fucose functions as a mediator of host–microbe, hybrid symbiosis,^91^ the infections by *H. pylori* and *V. cholera* occur on the epithelial barrier of the body under completely different topographical conditions when compared with malaria.

According to current reviews,^89^, ^90^, ^92^ the number of patients with blood type O(H), which apparently develop *H. pylori* infections more frequently, does not differ statistically significantly from that in which the ABO blood type does not play any role when compared by culture results. Rossez *et al*.^93^ have characterized the glycosylation pattern of gastric mucins and showed that about 80% of *O*‐glycans carried A, B, or H antigens and 30–50% expressed H and/or Le^b^ epitopes, which was most pronounced in blood group O individuals, whereas Boren *et al*.^94^ have shown by thin‐layer chromatography that *H. pylori* predominantly recognize Le^b^ and H‐active structures. Such observations have suggested that *H. pylori* infections might primarily be linked to the secretor status and that nonsecretors Le (a+ b–) are more susceptible. However, independent of *H. pylori* infections, individuals with blood group O and/or nonsecretors are especially prone per se to inflammatory bowel disease and gastric or duodenal ulcers but show more severe symptoms during these infections, whereas a variety of other factors are responsible for *H. pylori* virulence, as summarized in the current review by Kao *et al*.;^92^ *H. pylori* infection alone may be asymptomatic for a long time. Intriguingly, α1,2‐fucosyltransferase, which represents the basis for the synthesis of all ABO(H) epitopes and is secreted independently of the secretor status,^51^, ^52^ will also be produced by *H. pylori*. Thus, both host and pathogen produce the critical enzyme, and it is not clear whether pathogen adhesion is initiated by the host or the pathogen.

Although α1,2‐fucosyltransferase is produced by *H. pylori* and other Enterobacteriaceae,^95^ corresponding enzyme activity does not appear to occur in *V. cholera* strains and has not been reported for either *V. cholera* El Tor or *V. cholera* O139, which apparently show the strongest virulence and develop the most severe disease in human infections. The first studies that suggested a relation between cholera and ABO(H) blood groups were performed by Barua and Paguio,^96^ who reported a pronounced susceptibility to this infection in individuals with blood group O. This observation was substantiated by subsequent studies,^97^ while according to other reports one‐third of cholera patients are nonsecretors,^98^, ^99^ which is highly significant because the number of nonsecretors in all populations is not more than about 20%. However, blood group O does not increase the risk of being infected with *V. cholerae*:^100^ when ABO phenotypes were compared by culture results, the groups O and A were equivalent in *Vibrio*‐positive and *Vibrio‐*negative patients, but when the effect of cholera toxin (Ctx) was studied on enteroids, a significantly greater cyclic adenosine monophosphate response was shown in enteroids that were derived from blood group O stem cells.^101^ Thus, in the case of infections, individuals with group O more frequently develop life‐threatening disease and are more likely to be hospitalized with severe cholera^102^ when compared with non‐O blood groups.^103^

While in malaria tropica and blood group A individuals the parasite's entry into the patient's RBCs is probably determined by the functions of the soluble form of blood group A phenotype‐determining enzyme(s), the adhesion of *V. cholerae* toxin and its entry into the cytosol of the human blood group O(H) intestinal cells is currently explained by a different mechanism. Over the decades, several models have been developed that try to explain the adhesion of pathogenic Enterobacteriaceae or endotoxins to mammalian cell membranes. As early as 1947 Burnet and Stone^104^ discovered the “the receptor‐destroying enzyme,” a mixture of hydrolases dominated by a neuraminidase, associated with the secretion of adhesion proteins^105^ that cause pan‐agglutination and RBC rosette formation,^106^ which is known as the Hübener‐Thomsen Friedenreich phenomenon.^107^ The adherence of *V. cholerae* to isolated rabbit brush border membranes,^105^ human RBCs, and the lymphoid follicle epithelium of the intestine was (depending on the cellular HA type) inhibited by l‐fucose.^108^ These observations fit the established concepts. The principle is comprehensively described by Cooling^89^ and explained by means of a graphic, according to which the adhesion is thought to be primarily initiated by the pathogen, utilizing preformed fucosylated structures of the host; the adhesion of the Ctx holotoxin occurs via a complex binding process between the five toxin B subunits and the A subunit to cell surface receptors in the small intestine.^109^ However, this graphic may even imply a more active role of the host. Although a Galβ1‐3GlcNAcβ‐R structure has not been reported to occur on the capsule of *V. cholerae* and function as an acceptor for a host‐dominated α‐1,2‐l‐fucosylation that would best explain the adhesion of the pathogen to blood group O(H) intestinal cell surfaces, an adhesion might nevertheless be initiated via *O*‐fucosylation of serine/threonine residues of the Ctx subunits,^110^, ^111^, ^112^ which in this way get attached to the ganglioside GM1, Galβ1‐3GalNAcβ1‐4Galβ1‐4Glc‐ceramide. Again, the production and levels of soluble ABO(H)‐determining glycotransferases are independent of the secretor status,^51^, ^52^, ^113^ and it may be hypothesized that a host‐dominated adhesion precedes the established mechanisms, in which the pathogen may utilize the host cell machinery. This hypothetical α‐1,2‐l‐fucosylation occurs independently of ABO(H) blood groups, and in the non‐O blood groups A and B, the H‐underlying antigen quality will be reduced or neutralized by simultaneous phenotypic A and B glycosylation that occupies functional fucosyl residues, whereas the blood group O(H) individual will neither exhibit a natural, nonimmune anti‐H antibody against the resulting hybrid epitope under normal condition, nor produce an adaptive anti‐H‐reactive antibody due to clonal selection (Fig. 3). Significant production of adaptive and innate anti‐H antibodies is limited to the extremely rare, classical Oh *Bombay*‐type individual, lacking the normal, human‐specific α‐1,2‐l‐fucosyltransferase (FUT1/FUT2) functions^114^ and the respective fucosylations of plasma proteins.^115^ At present, the lowest prevalence of blood group O(H) worldwide occurs in the Ganges delta, where cholera has been endemic for centuries.^103^, ^116^, ^117^ Nevertheless, the evolutionary‐selective pressure that is exerted by the eukaryote *P. falciparum* and malaria tropica infections on blood group A populations is obviously much stronger. Consequently, blood group O(H), which offers the widest flexibility in adaptive and germline‐encoded immunity, has survived as the most frequently occurring blood group worldwide^118^ despite extensive historical cholera pandemics.^119^, ^120^, ^121^

## Conclusion

Naturally occurring immunoglobulins arise from various sources via different molecular pathways.^8^ While clonal selection protects mammalian species from the formation of environmentally induced adaptive, self‐reactive immunoglobulins, the human blood group A, B, and O(H) phenotypes additionally exclude the presence of a self‐reactive, germline‐encoded nonimmune IgM through identical glycosylation of cell surfaces and plasma proteins.^14^, ^15^ Consequently, phenotype‐specific susceptibility to infection by a special pathogen necessarily reduces or excludes natural immunological protection (Fig. 2). This implies that the phenotype‐determining glycotransferase might play the first role in determining the susceptibility of an individual to infection by a special pathogen; this enzyme both establishes contact with a complementary acceptor of the pathogen and reduces or excludes the presence of a phenotype‐specific antibody through glycosidic exclusion and/or accommodation of plasma proteins.^14^, ^15^ Again, the observations accumulated in the literature suggest that an infectious disease may be initiated via phenotypic accommodation between host and pathogen. In both the infection by the eukaryote *P. falciparum* and the prokaryote *V. cholerae*, the pathogen adhesion appears to be initiated by trans‐glycosylation via binding of the phenotype‐determining carbohydrate (GalNAc and/or l‐fucose) on hydrophilic amino acids from the pathogen peptide by *O*‐linkages. Thus, in the case of severe malaria in individuals with human blood group A, or cholera disease in blood group O(H), the contact between host and pathogen appears to be initiated through destructive cooperation between heterologous‐complementary carbohydrates and peptides. This implies that anti‐ABO(H)‐reactive immunoglobulins remain basically engaged in the control of those internal growth processes, from which most of them arise. Nevertheless, these immunoglobulins may nonspecifically or cross‐reactively protect the organism from environmental infections and pathogens that are not involved in their production, a phenomenon characteristic of the polyreactive defense proteins of invertebrates. The anti‐A/anti‐Tn‐cross‐reactive hemagglutinin emerging from the coat proteins of fertilized eggs of *Helix pomatia* 15 and lectins from other snails agglutinate *Staphylococcus aureus, E. coli, Listeria*, and several *Salmonella* species. This effect is likely due to *N*‐acetyl‐d‐galactosamine residues^122^ because all bacteria utilize this sugar in different metabolic pathways, which do not necessarily lead to complete antigenic structures. After all, the central immunological position of the human histo (blood) group O(H) remains evident in its evolutionary metabolic cross‐point between the regulation of trans‐species glycosylations on one side and its role as the structural basis of species‐intrinsic glycosylation or phenotype formation on the other side. This extensive molecular biological task explains its comprehensive presentation of both nonimmune IgM and adaptive IgM/IgG antibodies, which are mainly directed against all non‐O blood groups, involving their cross‐specific developmental and/or aberrant structures (Fig. 3), as early metazoan eukaryotic *O*‐glycans, Tn,^23^ and T^24^ antigens have been identified. These ancestral glycans arising from *O*‐glycosylations are used (with similar peptide backbones) by lower metazoans such as mollusks and the fruit fly *Drosophila melanogaster*,^123^, ^124^ and in the snail *Helix pomatia*, they are associated with the release of a hexamerically^125^ structured Tn‐complementary hemagglutinating defense protein. Hammarström^126^ showed that the binding patterns and the capacity of this molluscan protein to bind human blood group A RBCs are strikingly similar to those of the mammalian IgM molecule, giving rise to speculation regarding an evolutionary relationship with the mammalian nonimmune anti‐A‐reactive IgM molecule. While the germline of *H. pomatia* appears not to be burdened with A/B phenotypic accommodations, similar to the polyreactive ancestral IgM of human blood group O(H), this species and other snails never develop cancer. An even stronger immunity, involving a clinically relevant anti‐H‐reactive, complement‐binding IgM and most likely higher anti‐A/Tn and anti‐B/T levels than in blood group O(H), is exerted by the real blood group O or *Bombay*‐type Oh (h/h; se/se), according to a mutational meltdown of the H and Se gene functions on chromosome 19 encoding FUT1/FUT2. However, the extremely small population size of the classic *Bombay* type suggests an effect on reproductive health^115^ and demonstrates a negative evolutionary role. While the background of these mutations has been discussed controversially and studies on the functions of α‐1,2‐l‐fucosyltransferases in murine fertility^127^ may not be extrapolated to human phenotype development, primates use different pathways for critical fucosylation events,^128^ and human α‐1,2‐l‐fucosyltransferase genes are exclusively responsible for the formation and phenotypic expression of ABO(H) antigens.^129^ Thus, the formation of the Oh *Bombay* type represents the evolutionary opposite to that of the normal blood group O(H), which finally became most common blood group worldwide, as can be explained molecularly and immunologically. Although an internal selection process might, via a currently discussed survival advantage of the overall risk of developing cancer, together with silent ABO(H) incompatible pregnancies, have occurred over millions of years, in view of the global distribution of the ABO(H) blood groups, an evolutionarily selective infectious disease appears to be the major reason, and the selective pressure exerted by life‐threatening malaria tropica, which is deleterious to non‐O blood groups, explains the biological predominance of histo (blood) group O(H) much more. Again, while the life of malaria species exclusively depends on the biological altruism and host cell machinery of higher eukaryotic species, in the case of malaria tropica this trans‐species altruism is predominantly present in the human non‐O blood groups with self‐destructive behavior especially due to the metabolic complementarity of the eukaryotic parasite *P. falciparum* to blood group A phenotype or A‐like synthesis, excluding the corresponding immunological reaction.

## Competing interests

The author declares no competing interests.
